# The efficacy and safety of eravacycline compared with current clinically common antibiotics in the treatment of adults with complicated intra-abdominal infections: A Bayesian network meta-analysis

**DOI:** 10.3389/fmed.2022.935343

**Published:** 2022-09-16

**Authors:** Rui Meng, Xin Guan, Lei Sun, Zhengyang Fei, Yuxin Li, Mengjie Luo, Aixia Ma, Hongchao Li

**Affiliations:** ^1^School of International Pharmaceutical Business, China Pharmaceutical University, Nanjing, China; ^2^Center for Pharmacoeconomics and Outcomes Research, China Pharmaceutical University, Nanjing, China

**Keywords:** complicated intra-abdominal infections, eravacycline, randomized controlled trials, systematic review, network meta-analysis

## Abstract

**Background:**

Eravacycline is a novel, fully synthetic fluorocycline antibiotic for the treatment of adults with complicated intra-abdominal infections (cIAIs). However, the efficacy and safety of eravacycline compared with current clinically common antibiotics remain unknown.

**Objective:**

This study aims to compare the efficacy and safety of eravacycline and other clinically common antibiotics in China, including tigecycline, meropenem, ertapenem, ceftazidime/avibactam+metronidazole, piperacillin/tazobactam, imipenem/cilastatin, and ceftriaxone+metronidazole, for the treatment of adults with cIAIs and to provide a reference for clinical choice.

**Methods:**

The PubMed, Embase, Cochrane Library, and ClinicalTrials.gov databases were electronically searched to collect clinical randomized controlled studies (RCTs) comparing different antibiotics in the treatment of patients with cIAIs from inception to June 1, 2021. Two reviewers independently screened the literature, extracted data, and evaluated the risk of bias in the included studies.

**Results:**

A total of 4050 articles were initially retrieved, and 25 RCTs were included after screening, involving eight treatment therapies and 9372 patients. The results of network meta-analysis showed that in the intention-to-treat (ITT) population, the clinically evaluable (CE) population, and the microbiologically evaluable (ME) population, the clinical response rate of eravacycline was not significantly different from that of the other 7 therapies (*P* > 0.05). In terms of microbiological response rate, eravacycline was significantly better than tigecycline [tigecycline vs. eravacycline: RR = 0.82, 95%CI (0.65,0.99)], and there was no significant difference between the other 6 regimens and eravacycline (*P* > 0.05). In terms of safety, the incidence of serious adverse events, discontinuation rate, and all-cause mortality of eravacycline were not significantly different from those of the other 7 treatment therapies (*P* > 0.05).

**Conclusion:**

Based on the evidence generated by the current noninferiority clinical trial design, the efficacy and safety of eravacycline for the treatment of adults with cIAIs are not significantly different from those of the other 7 commonly used clinical antibiotics in China. In terms of microbiological response rate, eravacycline was significantly better than tigecycline. In view of the severe multidrug-resistant situation in China, existing drugs have difficulty meeting the needs of clinical treatment, and the new antibacterial drug eravacycline may be one of the preferred options for the treatment of cIAIs in adults.

## Introduction

Complicated intra-abdominal infections (cIAIs) in adults originate from abdominal organs and spread to the peritoneal or retroperitoneal space, causing peritonitis and abdominal abscesses. Intra-abdominal infections are the most common infectious disease among hospitalized patients (1–3), and the mortality rate varies greatly due to different factors, such as disease severity and the range of infecting pathogens. The mortality rate in patients with hospital-acquired cIAIs is significantly higher than that of community-acquired infections (10.4 vs. 2.8%) in China, and a higher mortality rate is prevalent in patients with cIAIs in the intensive care unit (ICU) (21.24–29.1%) (4–8). cIAIs can be combined with complications such as sepsis, septic shock, and multiple organ failure. Surgery combined with antibiotics is generally recommended as treatment in clinical practice (1).

Over the past century, great strides have been made to treat cIAIs with various drugs. However, with the widespread application of antibacterial drugs in clinical practice, especially their irregular use or overuse, antimicrobial resistance among pathogens continues to increase. Therefore, multidrug-resistant bacteria have become an important threat to human health around the world. The clinical efficacy of antibacterial drugs such as penicillin, cephalosporin and carbapenems in the treatment of cIAIs has been seriously affected in recent years due to the increasing drug resistance of carbapenem-resistant Enterobacteriaceae or bacteria that produce extended-spectrum-β-lactamases (ESBLs) (9). Studies have shown that the first-line empirical treatment failure rate of cIAIs is as high as 68.3% (10). Moreover, infections with multidrug-resistant bacteria will lead to a longer hospital stay and increase the risk of death for cIAI patients, which not only causes more serious damage to their health but also places a relatively heavy economic burden on their families and society (3, 11). For treatment of hospital-acquired and high-risk community-acquired cIAIs, the current commonly used antibiotics in China mainly include tigecycline, meropenem, ertapenem, ceftazidime/avibactam+metronidazole, piperacillin/tazobactam, imipenem/cilastatin, ceftriaxone+metronidazole and other monotherapies or combination therapies.

Eravacycline is a novel, fully synthetic fluorocycline antibiotic that has a broad antibacterial spectrum and can cover all common clinical pathogens except for *Pseudomonas aeruginosa*, including gram-negative and gram-positive aerobic and anaerobic strains (9, 12, 13). Furthermore, eravacycline show high sensitivity to drug-resistant bacteria, and it can therefore be used to treat infection with multidrug-resistant bacteria, such as bacteria producing ESBLs, carbapenem-resistant Enterobacteriaceae, and carbapenem-resistant *Acinetobacter baumannii*. The results of two phase III multicentre clinical randomized controlled trials (RCTs), IGNITE 1 and IGNITE 4 (12, 13), showed that the efficacy of eravacycline was not inferior to ertapenem and meropenem in patients with cIAIs. Based on this, eravacycline was approved by the United States and the European Union in 2018 for the treatment of adults with cIAIs. Although current evidence shows that the efficacy and safety of eravacycline are equivalent when compared with ertapenem and meropenem, considering that there are more treatment options for antibacterial drugs used in the clinical treatment of cIAIs in China and that we lack head-to-head clinical RCTs of these different antibacterial drugs, this study used a Bayesian network meta-analysis to compare the efficacy and safety of eravacycline and other clinically common antibiotics in the treatment of adults with cIAIs to provide evidentiary support and a reference for rational clinical drug use in China.

## Materials and methods

This study was conducted and performed in accordance with Preferred Reporting Items for Systematic Reviews and Meta-analyses (PRISMA) guidelines (Supplementary Table 1) (14).

### Literature search strategy

All clinical studies comparing the different antibiotics in the treatment of patients with cIAIs were identified through a systematic review of the literature in the PubMed, Embase, Cochrane Library, and ClinicalTrials.gov databases from inception to June 1, 2021. The following search terms were used: complicated intra-abdominal infection, clinical trial, randomized, efficacy, safety, eravacycline, tigecycline, ertapenem, meropenem, and ceftazidime/avibactam, among others. The reference lists of all retrieved articles were also reviewed to identify additional articles. The retrieval was taken in the form of a combination of subject words and free words. The final database-specific searches are presented in Supplementary Tables 2–5.

### Eligibility criteria

Studies meeting the following criteria were included: (1) type of the study, RCT; (2) target population, adult patients with cIAIs; (3) intervention, novel treatment drug eravacycline; (4) comparison, current commonly used antibiotics in China, including tigecycline, meropenem, ertapenem, ceftazidime/avibactam+metronidazole, piperacillin/tazobactam, imipenem/cilastatin, and ceftriaxone+metronidazole; (5) outcome, the primary clinical efficacy endpoint of this meta-analysis was clinical response assessed at the test-of-cure (TOC) visit based on a modified/intention-to-treat (ITT) analysis population; the secondary clinical efficacy endpoint were clinical response assessed at the TOC based on clinical evaluable (CE) and microbiological evaluable (ME) populations. Microbiological efficacy endpoints were microbiological response at the TOC. Safety outcomes were all-cause mortality, any adverse events (AEs) leading to discontinuation and serious AEs (≥ grade 3).

We excluded the following studies: (1) studies not in English; (2) duplicate studies; (3) systematic reviews, case observations, study protocols, lectures, conferences, and theses; (4) pooled analysis and post hoc analysis; (5) pharmacological, toxicology, molecular and animal experiments; (6) patients with any cancer; and (7) studies with no efficacy or safety outcomes.

### Selection of studies and data extraction

Two reviewers independently screened the literature, extracted data, and cross-checked the data. The titles and abstracts were screened to exclude obviously irrelevant literature, and the full texts were further screened to determine whether they were finally included. Discrepancies were resolved by consensus. The following data were extracted from the studies: (1) first author/year of publication; (2) characteristics of the target population: number of patients, age, Acute Physiology and Chronic Health Evaluation (APACHE) score and severity of infection; (3) interventions and comparisons; (4) duration of treatment; (5) key elements to assess risk of bias; and (6) outcome measures.

### Risk of bias assessment

We evaluated the methodological quality of the identified studies and cross-checked the results. The Cochrane Handbook for Systematic Reviews of Interventions version 5.1.0 was used to assess methodological quality. In terms of the assessment criteria, each trial was rated and assigned to one of the three following risks of bias: low risk, high risk or unclear. The evaluation items included random sequence generation (selection bias), allocation concealment (selection bias), blinding of participants and trial personnel (performance bias), blinding of assessor data (measurement bias), incomplete outcome data (follow-up bias), selective reporting of results (reporting bias) and other bias (15). Each evaluation item was divided into three levels: high risk of bias, low risk of bias and unclear risk.

### Statistical analysis

Bayesian network meta-analysis was performed by using R 3.6.1 software (all code was present in Supplementary Table 6). The relative risk (RR) and 95%CI were used as effect analysis statistics for binary classification efficacy outcome endpoints, and the odds ratio (OR) and 95%CI were used as effect analysis statistics for binary classification safety outcome endpoints. When the network plot had a closed loop, the node analysis method was used to test the consistency of the direct comparison and indirect comparison results of the treatments of the loop. At a *p* > 0.05, the direct comparison result was considered to be consistent with the indirect comparison result, and the consistency model (CM) was used for the network meta-analysis; otherwise, the inconsistency model (IM) was used. Each model was initially set up with 4 Markov chains for simulation, and the number of iterations was 200,000 times. The first 20,000 annealing times eliminate the influence of the initial value, and the degree of model convergence is diagnosed by the Potential Scale Reduction Factors (PSRF), which indicates that the model convergence is satisfactory if its value is close to 1. The publication bias was evaluated by funnel plot and Begg's test.

## Results

### Selection process and outcomes for the studies

**Figure 1:**
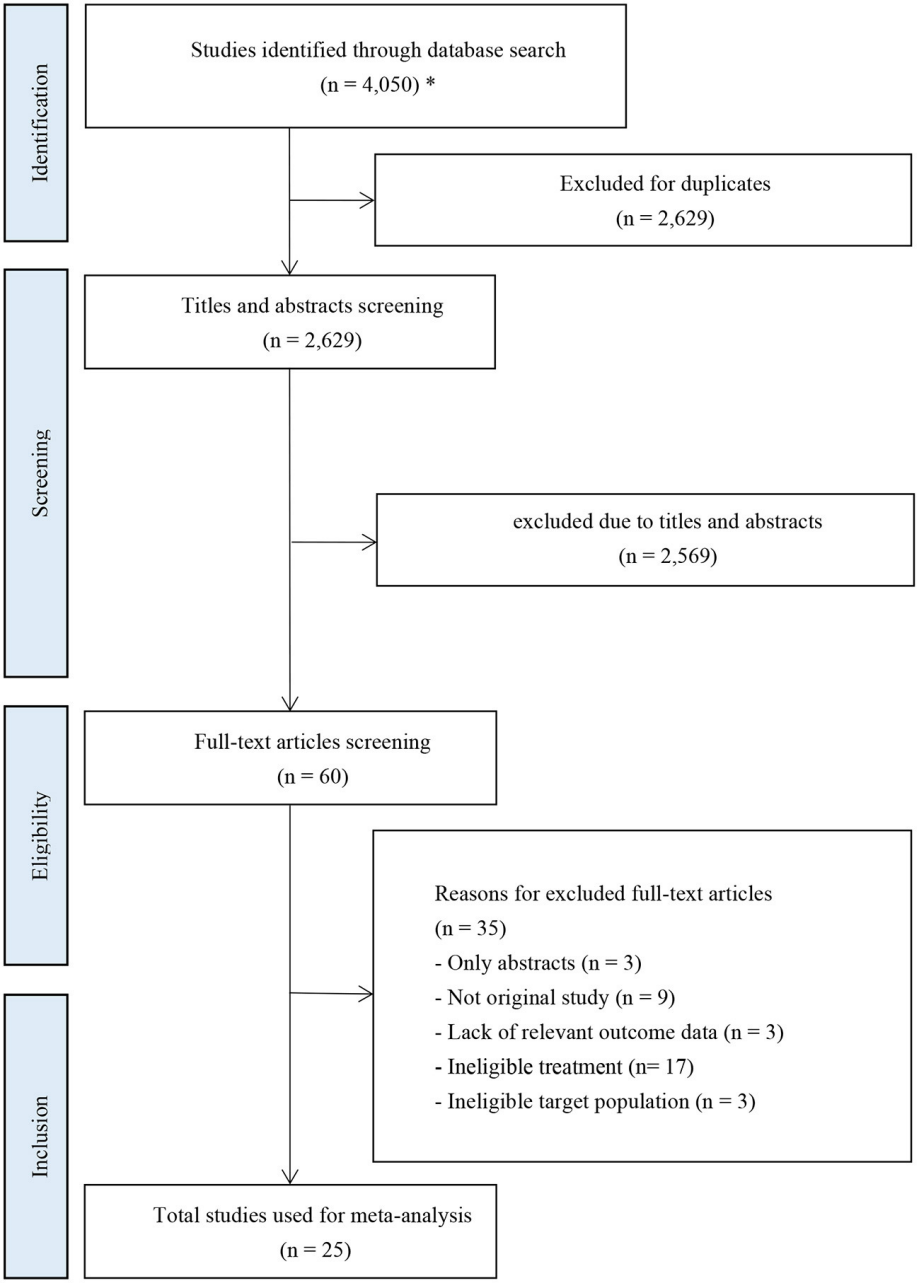
PRISMA flow diagram of study inclusion. ^*^The databases searched and the number of records retrieved are as follows: PubMed (*n* = 1,328); Embase (*n* = 1,936); Cochrane Library (*n* = 761); ClinicalTrials.gov (*n* = 25).

A total of 4,050 records were retrieved from the initial database search. Twenty-five RCT studies with a total of 9,372 patients were included after removing ineligible records. The selection process was shown in Figure 1.

### Characteristics and summary of risk of bias of included studies

**Figure 2:**
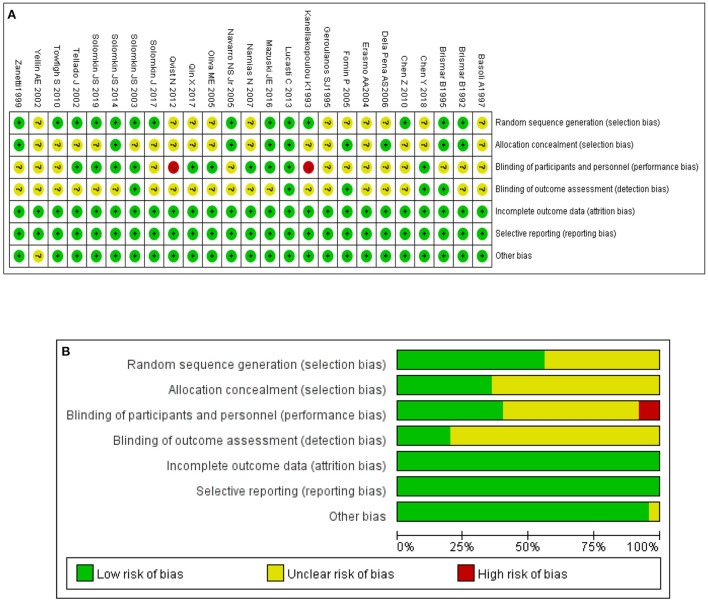
**(A)** Risk of bias summary. **(B)** Bar chart of risk of bias.

The basic characteristics of the included studies are listed in Table 1. Seventeen of the 25 RCTs were global multicentre studies. The assessment outcome of risk of bias is shown in Figures 2A,B. The figures show that 25 studies were of high overall quality, with 23 having a low or unclear risk of bias and two studies possibly having a risk of selection bias (16, 17).

**Table 1:** Characteristics of included studies.

**Author/year**	**Country/region**	**Numbers of patients (T/C)**	**Age (year) (T/C) (mean**±**SD)**	**APACHE II scores; (T/C) (mean** ±**SD)**	**APACHE II scores; (T vs C)(%)**	**Severity of infection**	**Intervention**		**Duration (Day) (T/C) (mean**±**SD)**	**Outcome endpoints**
							**Treatment**	**Comparator**		
Basoli et al. (16)	20 centers from Italy	101/100	54.4	6.4/5.9	≤ 10: 80.2% vs 82.6%; 11-20: 19.8% vs 13%; >20: 0 vs 2%	Mild to moderate (not life-threatening)	Ipmcil	Mem	6.7/7.2	
Brismar et al. (17)	6 centers from Sweden	69/65	52.9/54	NA	NA	NA	Tzp	Ipmcil	5.5/5.9	
Brismar et al. (18)	7 centers from Sweden	132/117	50.5/51.7	NA	≤ 10: 90.9% vs 89.7%; 11-20: 9.1% vs 9.4%; >20: 0 vs 0.9%	NA	Mem	Ipmcil	5/5	
Chen et al. (19)	47 centers from China	207/205	47.3 ± 17.74/8.7 ± 17.4	5.2 ± 3.38/5.4 ± 3.38	≤ 15: 100% vs 100%; >15: 0 vs 0%	NA	Tgc	Ipmcil	7.5/7.6	
Chen et al. (20)	China	97/102	46.8 ± 18.2/41.0 ± 16.7	5.1 ± 3.9 /4.1 ± 2.7	NA	Mild to moderate	Tgc	Ipmcil	5/6	
Dela Pena et al. (21)	48 centers worldwide	180/190	48/49	2/2 (median)	>10: 3.9 vs 4.2%	NA	Etp	Tzp	4-14	
Erasmo et al. (22)	China, Hong Kong, Malaysia, Korea, Philippines and Thailand	149/144	42.9 ± 18.3/41.3 ± 17.4	NA	NA	Moderate:65.4%; Severe:34.6%	Tzp	Ipmcil	5.6 ± 2.0/5.5 ± 2.1	
Fomin et al. (23)	94 centers from 27 countries in Europe, South Africa, Australia and Asia	404/413	48.3 ± 18.4/49.5 ± 18.0	6.44/ 6.41	NA	NA	Tgc	Ipmcil	7.7 ± 2.7/7.8 ± 2.7	
Geroulanos et al. (24)	12 centers from 6 countries in Europe	116/116	55/54	NA	NA	Mild: 15.9%; Moderate: 59%; Severe: 24.6%	Mem	Ipmcil	7.8/8.3	
Kanellakopoulou et al. (25)	Greece	32/30	NA	NA	NA	Moderate:100%	Mem	Ipmcil	7.7/8.6	
Lucasti et al. (26)	33 centers from Bulgaria, France, India, Lebanon, Poland, Romania, Russia and America	101/102	43.0 ± 15.9/42.6 ± 18.1	NA	≤ 10: 83.2% vs 83.3%; 11–25: 16.8% vs 16.7%	Less severely ill	Cazavi	Mem	6/6.5	
Mazuski et al. (27)	136 centers from 30 countries	529/529	49.8 ±17.5/50.3 ± 18.3	NA	≤ 10: 84.0% vs 83.0%; 11–30: 15.0% vs 15.3%; >30: 0.2% vs 0	NA	Cazavi	Mem	8.0 ± 3.3/8.3 ± 3.1	
Namias et al. (28)	61 centers from America	247/247	49.9/48.7	7.2 ± 4.3 / 6.4± 4.8	≤ 10: 83.7% vs 83.3%; >10: 16.3% vs 16.7%	NA	Etp	Tzp	7.0 ± 3.6/7.6 ± 4.0	
Navarro et al. (29)	53 centers from Latin America, Europe, Asia, Australia and South Africa	225/225	44/43.9	3/3 (median)	>10: 2.7% vs. 4.4%	NA	Etp	Ctrx	6/7	
Oliva et al. (30)	Multicenter worldwide	247/255	42.9 ± 18.0/43.1 ± 17.6	5.6 /5.5	NA	NA	Tgc	Ipmcil	8.1 ± 2.8/7.9 ± 2.7	
Qin et al. (31)	Centers from China, South Korea and Vietnam	214/217	48.5 ± 16.8/48.5 ± 17.4	NA	≤ 10: 93.9% vs. 92.6%; 11-30: 6.1% vs 7.4%	NA	Cazavi	Mem	6.9 ± 2.9/7.3 ± 2.8	
Qvist (32)	Centers from 19 countries in Europe, Asia, South Africa and the Middle East	232/235	48.55 ± 18.37/46.81 ± 18.38	6.22 ± 4.02 / 6.99 ± 4.70	NA	NA	Tgc	Ctrx	6.97 ± 3.01/6.93 ± 2.71	
Solomkin et al. (13)	66 centers from 11 countries	220/226	54.9 ± 17.14/55.4 ± 16.17	6.6 ±4.23 /6.8 ±3.94	NA	NA	Era	Etp	7.6 ± 2.8/7.6 ± 2.4	
Solomkin et al. (33)	57 centers from 18 countries	323/193	46.2 ± 19.0/45.4 ± 18.9	NA	0–4: 29% vs 28%; 5–9: 41% vs 46%; 10–14: 21% vs 18%; 15–19: 6% vs 6%; 20–24: 2% vs 1%; 25–29: 0.5% vs 0	NA	Etp	Tzp		
Solomkin et al. (12)	19 centers from 6 countries	57/30	42.1 ± 17.2/41.8 ± 17.6	6.0 ±3.8/6.1 ± 2.7	NA	NA	Era	Etp	6.3/6.2	
Solomkin et al. (9)	65 centers from 11 countries	195/205	50.3 ± 17.7/52.3 ± 18.3	6.6 ± 3.8 / 6.4 ± 4.0	NA	NA	Era	Mem	4-14	
Tellado et al. (34)	Multi-center worldwide	323/310	46.2 ± 19.0/45.4 ± 18.9	NA	0–4: 29.4% vs 29.6 %; 5–9: 41.1% vs 43.3%; 10–14:19.0% vs 18.6%; 15–19: 6.3% vs 7.2%	NA	Etp	Tzp	6/7	
Towfigh et al. (35)	53 centers from America, Canada and Latin America	236/231	48/48	NA	<10: 80% vs 81% 10–15: 16% vs 15% >15: 4% vs 4%	NA	Tgc	Ctrx	4-14	
Yellin et al. (36)	19 centers from America and Latin America	59/55	37.8 ± 18.1/41.1 ± 19.0	NA	0–4: 36% vs20% 5–9: 39% vs 56%; 10–14: 19% vs 18%; 15–19: 5% vs 2%; 20–24: 0% vs 2%	Mild to moderate	Etp	Ctrx	7.7 ± 4.3/8.8 ± 5.0	
Zanetti et al. (37)	Centers from Sweden	71/64	59.8 ± 18.5/60.0 ± 18.6	5.8 ±3.5/ 6.4 ±4.2	0–5: 48% vs 47%; 6–10: 41% vs 39%; 11–15: 10% vs 8%; 16–18: 1% vs 6%	Moderate	Mem	Ipmcil	9.5 ± 3.6/8.4 ± 2.9	

NA, not applicable; SD, standard deviation; Era, eravacycline; Etp, ertapenem; Mem, meropenem; Tgc, tigecycline; Cazavi, ceftazidime/avibactam + metronidazole; Tzp, piperacillin/tazobactam; Ctrx, ceftriaxone + metronidazole; Ipmcil, imipenem/cilastatin; Clinical response; Microbiological response; Mortality; any drug-related adverse events(AEs) leading to discontinuation; ⑤serious AEs(≥ grade 3).

### Convergence assessment and inconsistency test

The PSRF of the network meta-analysis model established according to all outcome indicators was close to 1, indicating that the model converged well and the outcomes were reliable. Since there were closed loops in the network diagrams of all outcome indicators, the node analysis method was used for the inconsistency test. The results showed no substantial difference (*P* > 0.05) between the direct comparison and indirect comparison of interventions in the loop, which met the requirements of consistency. Therefore, this study applied a consistency model to conduct a network meta-analysis of all outcome indicators.

### Network meta-analysis for clinical response

**Figure 3:**
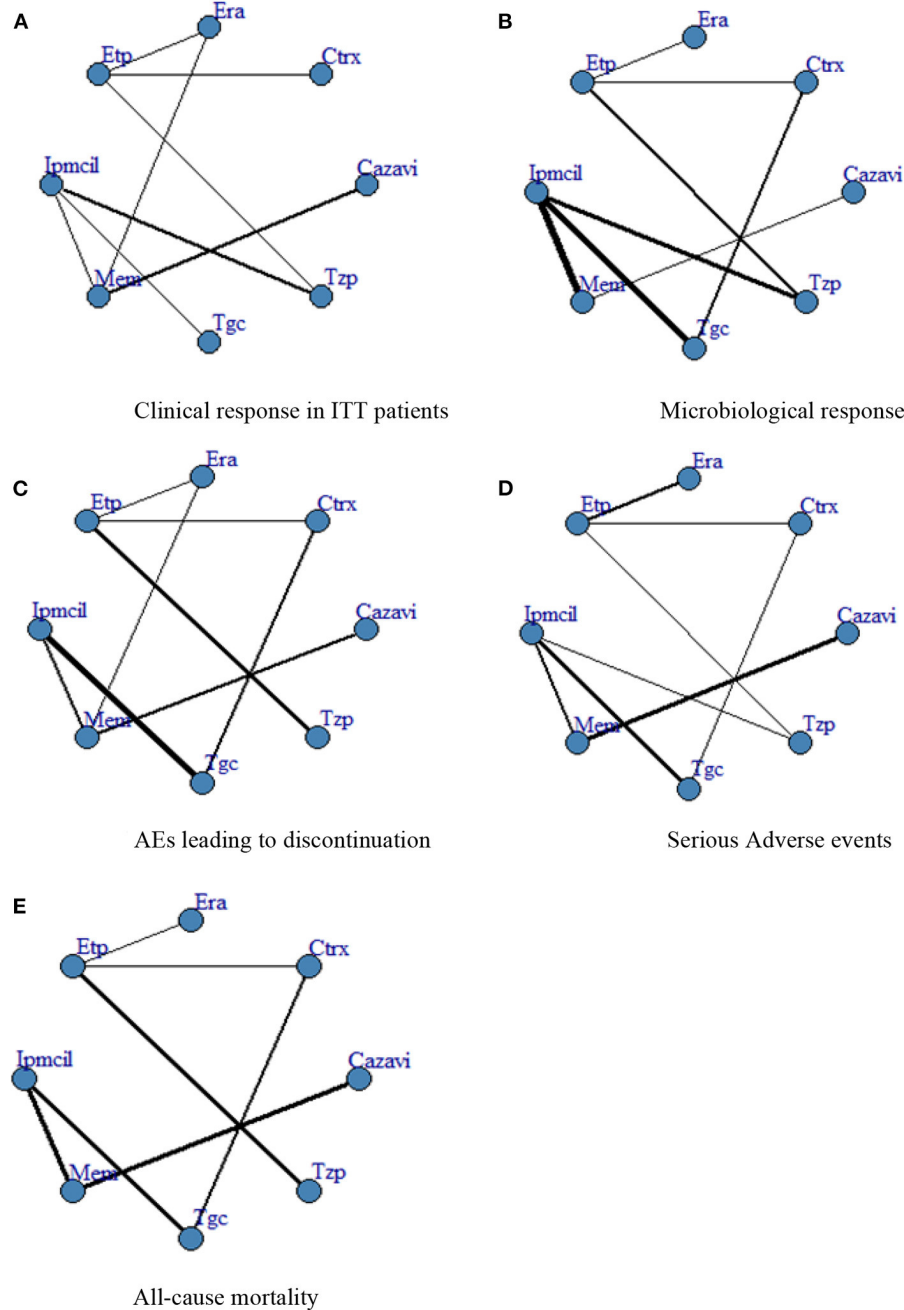
**(A–E)** Network plot. ITT, intention-to-treat; Era, eravacycline; Etp, ertapenem; Mem, meropenem; Tgc, tigecycline; Cazavi, ceftazidime/avibactam + metronidazole; Tzp, piperacillin/tazobactam; Ctrx, ceftriaxone + metronidazole; Ipmcil, imipenem/cilastatin.

**Figure 4:**
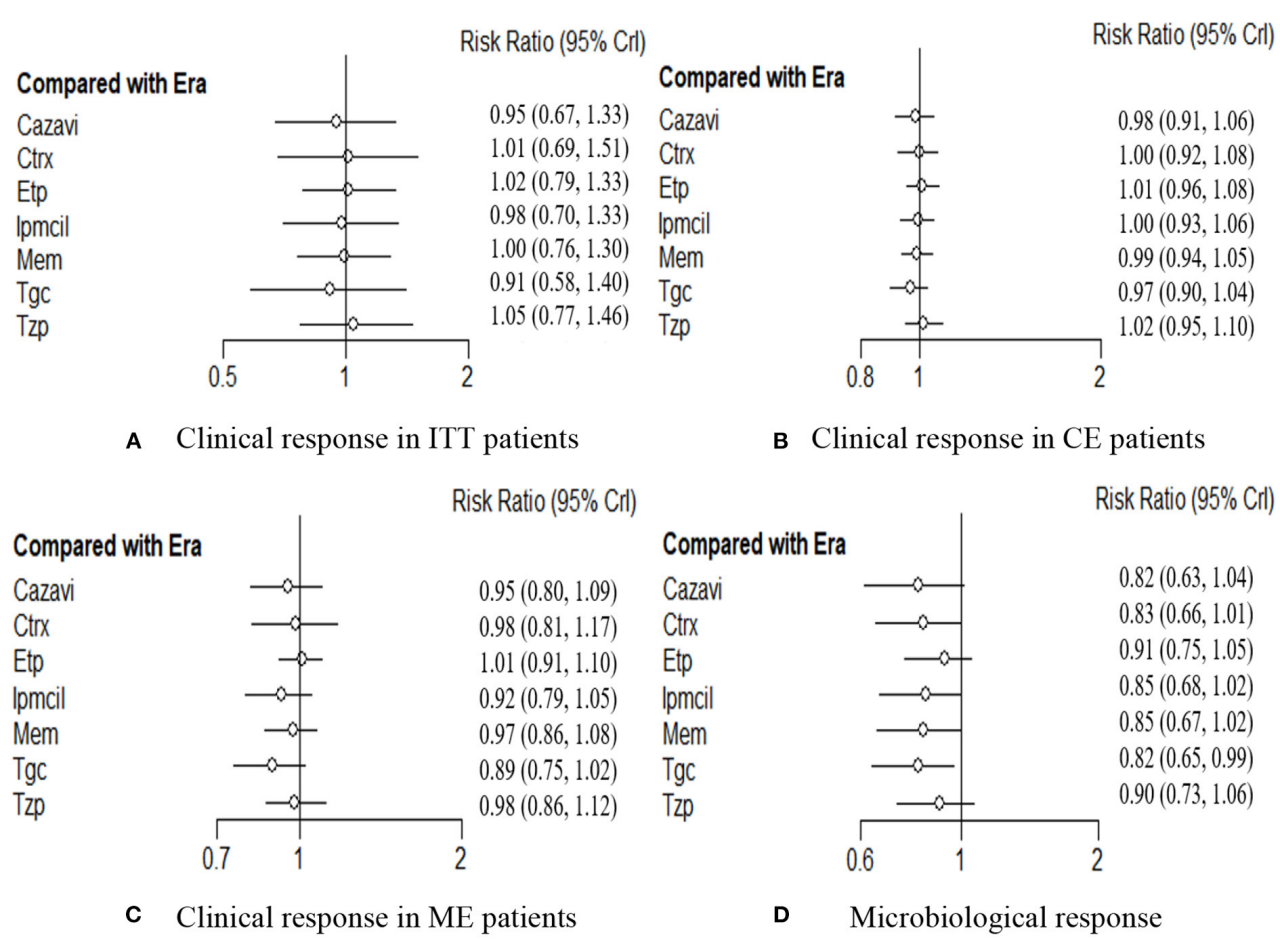
**(A**–**D)** Forest plot for efficacy endpoints. ITT, intention-to-treat; CE, clinical evaluate; ME, microbiological evaluable; Era, eravacycline; Etp, ertapenem; Mem, meropenem; Tgc, tigecycline; Cazavi, ceftazidime/avibactam + metronidazole; Tzp, piperacillin/tazobactam; Ctrx, ceftriaxone + metronidazole; Ipmcil, imipenem/cilastatin.

A total of 4,024 ITT patients in the 10 RCTs provided data for clinical response evaluation. The network plots are shown in Figure 3, the thicker the line in the plots is, the more direct comparative studies between the two interventions are. Eravacycline showed no significant differences compared with the other seven treatments. However, eravacycline achieved a higher absolute clinical response rate than tigecycline [Tgc vs. Era: RR = 0.91, 95%CI (0.58,1.40)], ceftazidime/avibactam plus metronidazole [Cazavi vs. Era: RR = 0.95, 95%CI (0.67,1.33)], imipenem/cilastatin [Ipmcil vs. Era: RR = 0.98, 95%CI (0.70,1.33)], meropenem [Mem vs. Era: RR = 1.00, 95%CI (0.76,1.30)] (Figure 4A). Meanwhile, no significant difference was observed between eravacycline and other comparators in CE patients (20 RCTs, 7,016 patients, Figure 4B) nor ME patients (15 RCTs, 3,756 patients, Figure 4C). The comparison outcomes of clinical response are presented in Supplementary Table 7.

### Network meta-analysis for microbiological response

Microbiological response was evaluated by 3,677 investigators in the 19 RCTs. Network meta-analysis showed significant efficacy of eravacycline compared with that of tigecycline [Tgc vs. Era: RR = 0.82, 95%CI (0.65,0.99)], but there was no significant difference from any of the other six comparators (*P* > 0.05, Figure 4D). The comparison outcomes of microbiological response are presented in Supplementary Table 8.

### Network meta-analysis for safety

**Figure 5:**
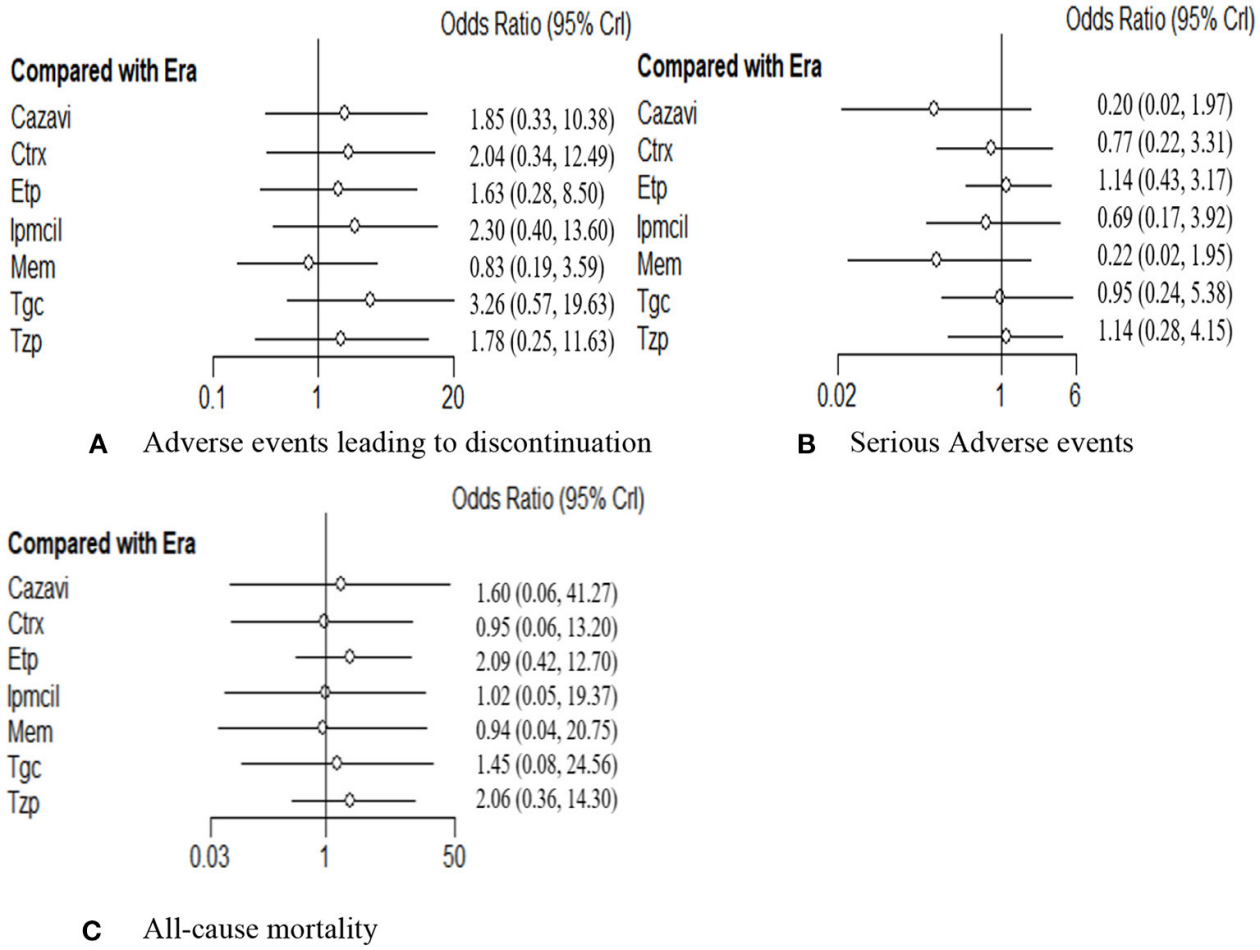
**(A**–**C)** Forest plot for safety endpoints. ITT, intention-to-treat; CE, clinical evaluate; ME, microbiological evaluable; Era, eravacycline; Etp, ertapenem; Mem, meropenem; Tgc, tigecycline; Cazavi, ceftazidime/avibactam + metronidazole; Tzp, piperacillin/tazobactam; Ctrx, ceftriaxone + metronidazole; Ipmcil, imipenem/cilastatin.

The rate of any AEs leading to discontinuation in 16 RCTs including 7,742 patients was evaluated. In addition to meropenem, eravacycline showed a lower adverse event discontinuation rate in treating cIAIs than the other six antibacterial drugs, but no significant difference was observed (Figure 5A). Additionally, differences did not reach statistical significance between eravacycline and the other drugs in the SAE rate (14 RCTs, 6,483 patients, Figure 5B) and all-cause mortality rate (16 RCTs, 7,766 patients, Figure 5C). The comparison outcomes of safety indicators are presented in Supplementary Table 9.

### Publication bias

**Figure 6:**
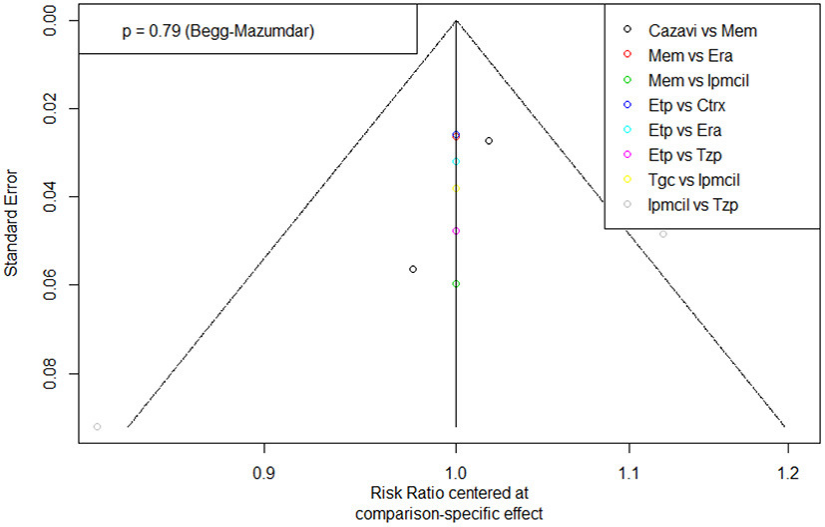
Funnel plot of Publication bias. Era, eravacycline; Etp, ertapenem; Mem, meropenem; Tgc, tigecycline; Cazavi, ceftazidime/avibactam + metronidazole; Tzp, piperacillin/tazobactam; Ctrx, ceftriaxone + metronidazole; Ipmcil, imipenem/cilastatin.

A funnel plot of clinical response for the ITT population was tested for publication bias and showed a generally symmetrical left-right distribution across each study point (Figure 6), which, combined with the results of Begg's test (*p* = 0.79), suggested a low likelihood of publication bias.

## Discussion

This study systematically searched RCTs of eravacycline and other clinically commonly used antimicrobial drugs in China for the treatment of adults with cIAIs and indirectly compared their efficacy and safety by Bayesian network meta-analysis. Based on the evidence generated from the current noninferiority clinical trial design, the clinical response rate of eravacycline for the treatment of adults with cIAIs was not statistically significantly different from that of the other seven clinically used antimicrobial drugs; the microbiological response rate was better than that of tigecycline, which was a statistically significant difference. In terms of safety, the incidence of serious adverse events, discontinuation rates, and all-cause mortality rates were also not statistically significantly different from the other seven treatment regimens for eravacycline.

Expert consensus on multidisciplinary management of intra-abdominal infections (1) recommends the selection of antimicrobial agents based on whether the patient is at high risk of treatment failure or death, the presence of serious infections, possible pathogens, the likelihood of multiple pathogenic infections, the local resistance rate of common causative organisms, and the risk of infection with drug-resistant organisms. Cephalosporins, carbapenems and enzyme inhibitor combinations are the first-line mainstream agents for empirical anti-infective therapy in hospital-acquired and high-risk community-acquired intra-abdominal infections, while ceftazidime/avibactam, tigecycline and eravacycline are used as appropriate therapies for target treatment. In clinical practice, drug selection remains difficult due to the lack of evidence of direct comparisons between different carbapenems, newer tetracyclines (tigecycline, eravacycline) and other antibacterial drugs.

In terms of efficacy, this study demonstrated no statistically significant difference between the clinical response rate of eravacycline and ceftriaxone, carbapenems and other commonly used antimicrobials. Lan (38) and Eljaaly (39) conducted meta-analyses of the efficacy and safety of eravacycline vs. meropenem and ertapenem for the treatment of cIAIs in 2019 and 2021, respectively, and their results showed that there was no statistically significant difference between the clinical response rate of eravacycline and the two carbapenem antimicrobials, which is consistent with the results of this study. However, in real-world clinical applications in China, resistance to cIAIs is serious, as the common pathogens of cIAIs are predominantly gram-negative bacilli. Taking ESBL enterobacteria commonly found in complicated abdominal infections as an example, the susceptibility of ESBL *Escherichia coli* infections to ceftriaxone was <1% in both cases, and the susceptibility of ESBL-producing *Klebsiella pneumoniae* to ceftriaxone and cefotaxime was only 1.7% (40). The emergence and prevalence of carbapenem-resistant strains has been caused by the high level of resistance of Enterobacteriaceae bacteria to the three generations of cephalosporins, further increasing the use of carbapenems. In China, the resistance rate of *Klebsiella pneumoniae* to carbapenem-resistant antimicrobials has rapidly increased from 3% in 2005 to more than 25% in 2021 and even up to more than 35% in some provinces (41). The results of a retrospective observational study conducted by Cancelli (42) showed that carbapenem-resistant patients treated with carbapenems had a lower clinical cure rate than nonresistant patients (resistant vs. nonresistant patients: 78.2 vs. 91.8%, *p* = 0.03). Thus, cephalosporins and carbapenems have poor clinical efficacy and high failure rates in the treatment of cIAIs, particularly in patients with resistant bacterial infections. Solomkin et al. [9, 12] demonstrated that the clinical cure rate of eravacycline in patients with ESBLs-producing pathogens was 100%, and in patients with ESBLs-producing Enterobacteriaceae was as high as 87.5%, with superior efficacy.

Eravacycline and tigecycline are new tetracycline antibacterial agents. The results of comparison of eravacycline with tigecycline show that in terms of efficacy, eravacycline has a higher numerical clinical response rate and a significantly better microbiological response rate than tigecycline. These results can be partly explained by the better antibacterial activity of eravacycline. Eravacycline optimizes its antibacterial activity through a unique modification of the core D-ring of tetracycline. Eravacycline has a 2- to 4-fold lower minimum inhibitory concentration MIC_90_ than tigecycline against common gram-negative bacteria in both the overall and multidrug-resistant populations (43, 44) and has better *in vitro* antibacterial activity. In terms of safety, the absolute values of AE discontinuation rates were lower for eravacycline than for tigecycline. Furthermore, the results of a pooled analysis of tigecycline all-cause mortality in clinical studies by McGovern (45) and Prasad (46) suggested that tigecycline may increase the risk of death. Therefore, compared to tigecycline, eravacycline has better efficacy in absolute value and better safety.

The key finding of this study is supported by the following evidence. First, the 25 RCTs included were of high quality and had a low risk of bias; thus, the results of network meta-analysis were highly credible and convincing. Second, the study was more comprehensive in its consideration of outcome indicators, considering the clinical response rates of the ITT, CE, and ME populations in terms of efficacy indicators and the rates of adverse discontinuation, mortality, and serious adverse events in terms of safety, making the conclusions more reliable and stable. Again, the drugs included in this study for the treatment of cIAIs were comprehensive, comparing the clinical efficacy and safety of the new antimicrobial drug eravacycline for the treatment of cIAIs with seven commonly used antimicrobial drugs in China. These results provide a reference for clinicians in terms of clinical decision-making and antimicrobial drug application.

This meta-analysis has certain limitations. First, as the definitions of clinical response rates and microbiological response rates for patients with cIAIs were not entirely consistent across RCTs, which may have led to some bias in the results of the network meta-analysis. Second, considering the lack of high-quality cohort studies related to cIAIs antibacterial drugs in China, we can only make recommendations and references based on published RCTs worldwide. Therefore, the results need to be verified by further RCT studies in Chinese patients. Third, due to the absence of patient's resistance data in the original studies included, we were unable to perform a meta-analysis of the efficacy for different baseline pathogens. but published studies have demonstrated the severity of cephalosporin- and carbapenem-resistant strains, and the superior efficacy of eravacycline against extended-spectrum beta-lactamase (ESBL)-producing pathogens has also been confirmed [9, 12, 40–42]. Fourth, considering the increasing antimicrobial resistance, the efficacy of the antibiotics might change throughout years, which introduces some uncertainty to the results. Finally, due to limited data acquisition, the baseline characteristics of patients could not be matched to be completely consistent, and there were only three studies related to eravacycline in the included studies. Further direct comparative studies are needed to confirm the results.

## Conclusions

Based on the evidence generated by the current noninferiority clinical trial design, the efficacy and safety of eravacycline for the treatment of cIAIs in adults was not statistically significantly different from the other seven clinically commonly used antimicrobial drugs. In terms of microbiological response rates, eravacycline was statistically significantly better than tigecycline. In view of the severe resistance situation of penicillin, cephalosporins and carbapenems in China and the difficulty of existing drugs to meet clinical treatment needs, the new antimicrobial drug eravacycline may be one of the preferred options for the treatment of cIAIs in adults.

## Data availability statement

The original contributions presented in the study are included in the article/Supplementary material, further inquiries can be directed to the corresponding authors.

## Author contributions

RM, XG, AM, and HL: conception of the study. RM, LS, and ZF: literature search and data extraction. RM, XG, LS, and ZF: statistical analysis. RM, YL, and ML: checked the data and drafting the manuscript. AM and HL: revising and completion of final work. All authors reviewed and approved the final version.

## Conflict of interest

The authors declare that the research was conducted in the absence of any commercial or financial relationships that could be construed as a potential conflict of interest.

## Publisher's note

All claims expressed in this article are solely those of the authors and do not necessarily represent those of their affiliated organizations, or those of the publisher, the editors and the reviewers. Any product that may be evaluated in this article, or claim that may be made by its manufacturer, is not guaranteed or endorsed by the publisher.
